# A lipid metabolite lipidomics assay for prediction and severity evaluation of rotator cuff injury

**DOI:** 10.3389/fnut.2022.1000947

**Published:** 2022-09-23

**Authors:** Hongjie Huang, Dina Jiesisibieke, Xiang Zhou, Zhu Zhang, Xiaoning Duan, Xu Cheng, Zhenxing Shao, Jianquan Wang, Xin Zhang

**Affiliations:** ^1^Institute of Sports Medicine, Beijing Key Laboratory of Sports Injuries, Peking University Third Hospital, Beijing, China; ^2^School of Clinical Medicine, Peking University Health Science Center, Beijing, China

**Keywords:** rotator cuff, musculoskeletal disorders, lipidomics, dyslipidemia, serum lipids

## Abstract

**Objective:**

Rotator cuff injury can be caused by local inflammation and fibrosis of musculotendinous cuff. Hypercholesterolemia can lead to physiological changes of rotator cuff that resemble rotator cuff injury. However, the relationship between lipid metabolism and rotator cuff injury and its potential pathological mechanism remain unclear. Herein, we aimed to investigate the correlation between the plasma lipidome, rotator cuff injury, and successive fatty infiltration pathology, and hoped to identify biomarkers for predicting higher risk or higher severity rotator cuff injury by assessing metabolic perturbations and dyslipidemia using lipidomics.

**Methods:**

We quantitatively analyzed 60 lipids species of seven lipids classes and subclasses from 66 subjects using lipidomics. Subjects were divided into four groups: (1) normal rotator cuff with normal clinical routine serum lipid test results (NN group = 13); (2) normal rotator cuff with abnormal clinical routine serum lipid test results (NA group = 10); (3) rotator cuff tear with normal routine serum lipid test results (RN group = 30); (4) rotator cuff tear with abnormal routine serum lipid test results (RA group = 13). Independent-sample *t*-tests and Kruskal-Wallis tests were used to compare lipid metabolite levels in serum between different groups in patients with rotator cuff tears. The orthogonal partial least squares-discriminant analysis (OPLS-DA) model was used to verify the ability of five lysophosphatidylcholines (LPCs) to distinguish rotator cuff injuries. In the rotator cuff tear group, magnetic resonance imaging (MRI) was used to classify fatty infiltration according to Goutallier's classification. Kruskal-Wallis tests were used to analyze molecular differences between high-grade (grade 3–4) and low-grade (grade 0–2) fatty infiltration groups. Receiver operator characteristic (ROC) curves were drawn for each diagnostic method *via* different metabolites. The area under the curve (AUC), cutoff, specificity, sensitivity, and accuracy of each diagnostic criterion were calculated.

**Results:**

Our results showed that some rotator cuff injury patients yielded unique lipidomic profiles. Based on Kruskal-Wallis tests, our results showed significant differences in three lipid molecules, 17:1 Lyso PI, 18:0–22:6 PE, and 18:3 (Cis) PC, among all four groups independent of clinical blood lipid levels. Also, independent of clinical blood lipid levels, two lipid molecules, 22:0 Lyso PC and 24:0 Lyso PC, were significantly different between the two groups based on Independent sample *t*-tests. Kruskal-Wallis test results showed that in the rotator cuff tear group, two metabolites (24:0 SM and 16:0 ceramide) differed between high-grade and low-grade fatty infiltration. The AUC values for 22:0 Lyso PC, 24:0 Lyso PC, 18:0–22:6 PE, 24:0 SM, and 16:0 ceramide were 0.6036, 0.6757, 0.6712, 0.8333, and 0.8981, respectively.

**Conclusion:**

The results provide insight into how the metabolic mechanisms associated with dyslipidemia impact rotator cuff diseases. Five lipid molecules, 17:1 Lyso PI, 18:0–22:6 PE, 18:3 (Cis) PC, 22:0 Lyso PC, and 24:0 Lyso PC, were closely related to rotator cuff tear based on two statistical analysis methods, independent of clinical routine serum lipid test results, which indicates that lipidomics assays are more sensitive than conventional lipid tests, and more suitable for studying rotator cuff lipid metabolism. In addition, two lipid metabolites, 24:0 SM and 16:0 ceramide, are potentially useful for predicting fatty infiltration severity. Further research with a larger number of samples is needed to verify whether these two metabolites can serve as potential markers of severe fatty infiltration. The findings illuminate how metabolic mechanisms associated with dyslipidemia affect rotator cuff disease.

## Introduction

Lipid metabolism plays a crucial role in cellular physiological function (1, 2). Dysregulation of lipid metabolism contributes to various diseases such as atherosclerosis and stroke (3). Previous studies have shown that metabolic disorders are closely related to the progression of tendon injury, among which dyslipidemia, hyperuricemia, diabetes, obesity and some rare congenital metabolic disorders are often related to tendon degeneration (4). Nevertheless, the potential relationships between tendon/muscle disorders and dyslipidemia remain unclear. Current studies suggest that hyperlipidemia is associated with tendon pathology (5). Of note, Achilles's tendon xanthomas and rotator cuff disease are considered to be related to dyslipidemia (6, 7). Rotator cuff tear is one of the more common tendon disorders, often affecting older people, and predicted to affect 1 in 4 adults in the USA (8). The influence of the lipid profile on rotator cuff tear is manifold. Firstly, numerous extrinsic and intrinsic factors associated with an increased risk of rotator cuff tear and hyperlipidemia have been proposed as a mechanism of intrinsic rotator cuff pathology (9, 10). Dyslipidemia is probably involved in the mechanism of musculotendinous cuff weakening, resulting in an increased risk of tearing (11). Secondly, hyperlipidemia may influence the severity of injury. Park et al. (12) demonstrated a significant association between preoperative hypo high-density lipoprotein cholesterolemia and increased preoperative tear size (12). Thirdly, dyslipidemia has been identified as a risk factor for patient outcomes following rotator cuff repair, including postoperative complications, biomechanical healing failure which causes shoulder stiffness and other shoulder joint function decline, and re-tears (13, 14). Laboratory studies on a rabbit model suggested that hypercholesterolemia had a deleterious effect on fatty infiltration and the quality of tendon-to-bone repair, which can partially explain the worse outcomes and higher re-tear rates in some patients after arthroscopic rotator cuff repair (15).

However, direct and clear correlations between serum lipid levels and rotator cuff tear have not been confirmed. Studies by Abboud and Kim (7) showed higher total cholesterol, triglyceride, and low-density lipoprotein cholesterol concentrations in patients with rotator cuff tendon tears than controls, while their high-density lipoprotein cholesterol levels were lower (7). By contrast, other studies showed no significant difference in serum lipids between rotator cuff tear patients and those without a tear (10, 16). Most previous studies has focused on dyslipidemia phenotypes or individuals, and metabolic perturbations associated with dyslipidemia in patients suffering from rotator cuff tears remain poorly understood.

The purpose of this study was to investigate the association of the plasma lipidome with rotator cuff tear and successive fatty infiltration pathology. We also performed lipid metabolomics analysis to identify possible high-sensitivity markers that can be used to diagnose early dyslipidemia and rotator cuff tear. The findings lay a foundation for revealing associations between lipid metabolism and rotator cuff pathology.

## Materials and methods

### Subjects and data

This study was approved by the Ethics Committee of Peking University Third Hospital, Beijing, China (Approval number IRB00006761-M2021464). Our study included 43 patients who underwent rotator cuff surgery in the Institute of Sports Medicine, Peking University Third Hospital, during February 2022 to June 2022. A total of 23 people who underwent other surgery (e.g., arthroscopic meniscus plasty, arthroscopic acetabuloplasty, femoral neck osteoplasty) at our institute during the same period were recruited concurrently. Among the above-mentioned patients, 66 patients were divided into four groups based on clinical lipid levels and presence/absence of rotator cuff tear as follows: (1) normal rotator cuff with normal clinical routine serum lipid test results (NN group = 13); (2) normal rotator cuff with abnormal clinical routine serum lipid test results (NA group = 10); (3) rotator cuff tear with normal routine serum lipid test results (RN group = 30); (4) rotator cuff tear with abnormal routine serum lipid test results (RA group = 13). General data, blood lipid levels, erythrocyte sedimentation rate (ESR), C-reactive protein (CRP), and Barthel score of subjects were collected through the hospital medical record system. In the rotator cuff tear group, magnetic resonance imaging (MRI) was used to classify fatty infiltration according to Goutallier's classification (17).

### Metabolic measurements

Participants were divided into groups according to traditional blood lipid levels, in which normal blood lipids were defined as abnormal levels of at least two of the following four lipids: total cholesterol (TC) ≥ 5.18 mmol/L; triglyceride (TG) ≥1.70 mmol/L; high-density lipoprotein cholesterol (HDL-C) ≤1.04 mmol/L; low-density lipoprotein cholesterol (LDL-C) ≥3.64 mmol/L. Plasma samples from all patients included in the study were collected by the laboratory of the Peking University Third Hospital.

Lipomics data were quantified by the laboratory using Ekspert Ultra LC 100 and AB SCIEX Triple TOF 5,600 instruments. For the sample pretreatment process, plasma was frozen at −80°C, thawed in a refrigerator at 4°C, 10 μL was mixed with 10 μL of mixed internal standard solution, 10 μL of 0.9% NaCl, 100 μL of chloroform-methanol (2:1) extract was added, and samples were placed in a refrigerator at 4°C with 20 μL scroll for 30 min. After centrifugation at 7,800 g for 3 min, a 1 mL syringe was used to transfer the lower layer into a 0.5 mL EP tube and 40 μL was transferred into another EP tube, dried using a nitrogen stream, and placed in a freezer at −20°C until needed. Before injection, samples were dissolved in 50 μL of acetonitrile-isopropanol (1:1) with vortexing for 60 s.

Lipidomic analysis was performed using a Waters XBridge Peptide BEH C18 column (3.5 μm, 2.1 × 100 mm), a Phenomenex C18 column (4 × 2.0 mm), and an Allegra 64R centrifuge (Beckman Coulter, Brea, CA, USA). Referring to the lipid standard database, lipids in samples were qualitatively determined by PeakView1.2 software, then quantified by MultiQuant2.1 (Admei Company, Liaoning, China).

### Fatty infiltration assessment

All patients underwent MRI analysis preoperatively. Examinations were performed using a GE Discovery MR750 3.0 T System (PrizMed Imaging, Unit A Willowick, OH, USA). Researchers studied T2-weighted images with fat suppression in the coronal and sagittal planes, T2-weighted images without fat suppression in the coronal and sagittal planes, and T1-weighted images without fat suppression in the coronal plane.

In the sagittal oblique views in T1-weighted MRI, the outermost level where the scapula joins the scapula body (standard level) and its contiguous two layers were used to assess fatty infiltration level. Patients without any of these layers were excluded when assessing fatty infiltration.

**Figure 1:**
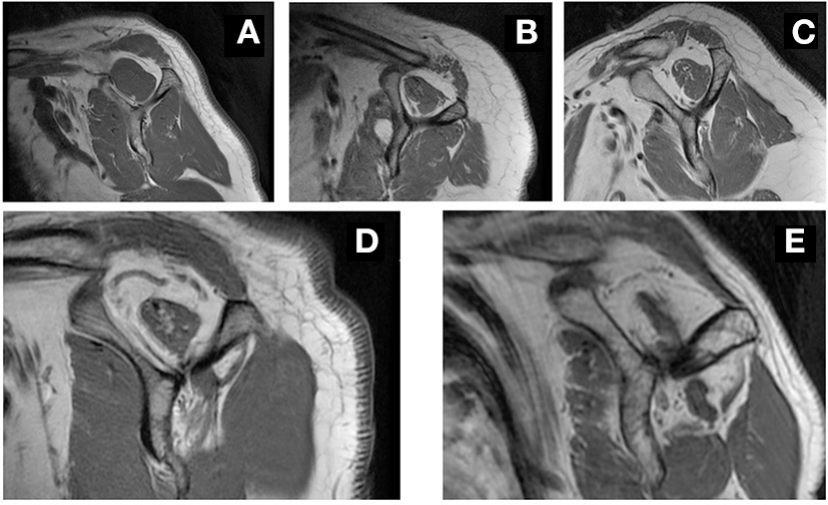
Representative MRI image in grading fatty infiltration. Sagittal oblique views in T1-weighted MRI, showing grade 0 **(A)**, grade 1 **(B)**, grade 2 **(C)**, grade 3 **(D)** and grade 4 **(E)** fatty infiltration of the supraspinatus muscle.

Two well-trained observers analyzed all images and classified fatty infiltration of the supraspinatus muscle layers using Goutallier's classification (17). An absence of intramuscular fat was classified as stage 0; the presence of some fatty streaks was classified as stage 1; the presence of substantial fat less than the amount of muscle was classified as stage 2; the presence of a quantity of fat roughly equal to the muscle volume was classified as stage 3; the presence of more fat than muscle was classified as stage 4. Stages 0, 1, and 2 were classified as low-grade fatty degeneration, and stages 3 and 4 were categorized as high-grade fatty degeneration (18) (Figure 1).

### Statistical analyses

All analyses were performed with R version 4.2.1. Independent-samples *t*-tests and Kruskal-Wallis tests were used to compare lipid metabolite levels in serum between different groups of patients with rotator cuff tears. The Benjamini and Hochberg method was used to modify the overall *p*-value. Kruskal-Wallis tests were also used to analyze molecular differences between high-grade (grade 3–4) and low-grade (grade 0–2) fatty infiltration groups, and *p* < 0.05 was considered statistically significant. For each metabolite, we drew ROC curves to estimate the diagnostic power. The diagnostic thresholds of the metabolites were calculated and used in further diagnostic tests. Sensitivity, specificity, and accuracy were calculated for each diagnostic criterion.

## Results

The demographic characteristics of the study population are summarized in Table 1. Rotator cuff tear groups were significantly older than normal groups and had higher body weight (*p* < 0.001). No significant differences were observed in age, weight, body mass index (BMI), Barthel score, CRP, or ESR between the four groups.

**Table 1:** Demographic and clinical parameters^a^ of the study population.

	**Age (years)**	**Gender**	**Weight (kg)**	**BMI (kg/m^2^)**	**Barthel score**	**TC** **(mmol/L)**	**TG (mmol/L)**	**LDL-C (mmol/L)**	**HDL-C (mmol/L)**	**CRP (mg/L)**	**ESR (mm/h)**
**Rotator cuff tear group (*****N*** **=** **43)**	56.77 ± 11.38	Male: 48.8% (21/43)	71.91 ± 20.67	25.23.13 ± 3.46	77.56 ± 3.56	5.04 ± 1.17	1.49 ± 0.92	2.94 ± 0.89	1.24 ± 0.29	0.42 ± 0.87	9.33 ± 9.99
		Female: 51.2% (22/43)									
RN group (*N* = 30)	57.17 ± 11.85	Male: 50.0% (15/30)	73.87 ± 22.44	25.08 ± 2.66	77.59 ± 3.44	4.49 ± 0.85	1.37 ± 0.67	2.51 ± 0.61	1.21 ± 0.26	0.48 ± 1.04	9.27 ± 11.47
		Female: 50.0% (15/30)									
RA group (*N* = 13)	55.85 ± 10.60	Male: 46.2% (6/13)	67.38 ± 15.72	25.55 ± 4.97	77.50 ± 3.99	6.26 ± 0.79&&&	1.77 ± 1.33	3.91 ± 0.63&&&	1.31 ± 0.35	0.27 ± 0.14	9.46 ± 5.62
		Female: 53.8% (7/13)									
**Normal group (*****N*** **=** **23)**	45.91 ± 10.35***	Male: 56.5% (13/23)	67.96 ± 11.53***	23.89 ± 3.08	75.45 ± 1.47	5.53 ± 1.54	1.80 ± 1.14	3.26 ± 1.11	1.32 ± 0.37	0.57 ± 1.64	9.59 ± 6.67
		Female: 43.5% (10/23)									
NN group (*N* = 13)	43.46 ± 10.43	Male: 61.5% (8/13)	68.08 ± 11.54	23.91 ± 3.20	75.00 ± 0.00	4.70 ± 0.47	1.39 ± 0.74	2.77 ± 0.46	1.28 ± 0.25	0.19 ± 0.09	8.08 ± 4.96
		Female: 38.5 (5/13)									
NA group (*N* = 10)	49.10 ± 9.84	Male: 50.0% (5/10)	67.80 ± 12.14	23.87 ± 3.09	76.11 ± 2.20	6.60 ± 1.80**##**	2.33 ± 1.37**##**	3.89 ± 1.40**##**	1.36 ± 0.50	1.07 ± 2.46	11.40 ± 8.18
		Female: 50.0% (5/10)									

a All values are presented as mean ± standard deviation (SD). TG, triglyceride; TC, total cholesterol; LDL-C, low-density lipoprotein cholesterol; HDL-C, high-density lipoprotein cholesterol; NN, normal rotator cuff with normal clinical routine serum lipid test results; NA, normal rotator cuff with abnormal clinical routine serum lipid test results; RN, rotator cuff tear with normal routine serum lipid test results; RA, rotator cuff tear with abnormal routine serum lipid test results. Traditional abnormal clinical plasma lipid levels were defined as abnormal levels of at least two of four lipids (TG ≥1.70 mmol/L; TC ≥5.18 mmol/L; L-DLC ≥3.64 mmol/L; HDL-C ≤1.04 mmol/L).

*** Rotator cuff tear group vs. normal group, p < 0.001;

## NN vs. NA, p < 0.05;

&&& RN vs. RA, p < 0.001.

### Clinical plasma lipid levels

Clinical plasma lipid levels of the study population are summarized in Table 1. There was no significant difference in clinical plasma lipid levels between rotator cuff tear and normal groups. Levels of TC differed significantly with clinical plasma lipid levels in normal groups (*p* < 0.05). Similarly, in the rotator cuff tear groups, levels of TG and LDL-C were significantly different from clinical plasma lipid levels (*p* < 0.001).

### Lipids that discriminate rotator cuff tear

Kruskal-Wallis tests were used to compare the concentrations of 60 lipid species of seven lipid classes, and independent sample *t*-tests were used for subclasses between rotator cuff tear and normal groups with different clinical lipid levels (Table 2). The results revealed statistically significant differences in five lipid metabolites; 17:1 Lyso PI, 18:0–22:6 PE, and 18:3 (Cis) PC were significantly different between the four groups (*p* < 0.05) based on Kruskal-Wallis tests, and 22:0 Lyso PC and 24:0 Lyso PC were different between NN and RN groups based on independent sample *t*-tests (*p* < 0.05). Levels of 17:1 Lyso PI were significantly higher in the normal rotator cuff group and the normal clinical plasma lipid (NN) group than the other three groups (*p* = 0.014). A significantly lower level of 18:0–22:6 PE was observed in the rotator cuff tear group with normal clinical plasma lipid levels (RN) than the other groups (*p* = 0.020). The level of 18:3 (Cis) PC was significantly higher in the rotator cuff tear group with abnormal clinical plasma lipid levels (RA) than others (*p* = 0.036). Regardless of clinical plasma lipid levels, there were significant differences in the levels of some lipid species between rotator cuff tear patients and people with normal rotator cuff. In the groups with traditional normal clinical plasma lipid levels, significantly higher levels of 22:0 Lyso PC and lower levels of 24:0 Lyso PC were observed in the rotator cuff tear group than in the normal group (*p* < 0.05).

**Table 2:** Lipids that discriminate rotator cuff tear^a^.

**Variable**	**NN group**	**RN group**	***p*-Value**	**NA group**	**RA group**	***p*-Value**	**Overall**	**BH-adjusted**
	**(nmol/l)**	**(nmol/l)**	**(NN vs. RN)**	**(nmol/l)**	**(nmol/l)**	**(NA vs. RA)**	***p*-Value**	***p*-Value**
Egg Lyso PC	664,069.33 ± 303,137.83	675,972.29 ± 308,426.16	0.908	604,912.4 ± 229,696.43	635,493 ± 232,678.84	0.756	0.89	0.924
14:0 Lyso PC	3,190.79 ± 1,553.63	2,849.21 ± 1,325.27	0.465	2,627.65 ± 1,054.13	3,127.91 ± 1,148.15	0.296	0.58	0.908
15:0 Lyso PC	2,386.91 ± 961.19	2,594.18 ± 1,317.71	0.613	2,543.58 ± 1,023.07	2,385.25 ± 537.99	0.636	0.99	0.995
17:0 Lyso PC	5,264.95 ± 3,356.48	5,709.61 ± 3,955.23	0.726	5,866.34 ± 2,696.5	5,148.15 ± 1,690.49	0.442	0.87	0.924
18:0 Lyso PC	146,146.33 ± 79,241.13	139,632.04 ± 66,055.86	0.781	132,419.33 ± 63,260.41	147,150.59 ± 73,769.7	0.619	0.88	0.924
18:1 Lyso PC	66,267.42 ± 21,048.72	71,140.34 ± 41,043.53	0.689	58,944.16 ± 17,540.67	60,333.12 ± 22,972.73	0.875	0.79	0.924
20:0 Lyso PC	654.65 ± 123	676.49 ± 326.37	0.817	623.5 ± 196.11	686.64 ± 291.63	0.563	0.89	0.924
22:0 Lyso PC	164.21 ± 11.95	179.71 ± 36.32	0.043##	176.91 ± 36.11	171.58 ± 20.03	0.656	0.61	0.914
24:0 Lyso PC	2,618.28 ± 1,080.31	1,903.3 ± 951.69	0.036##	2,194.68 ± 668.67	1,866.57 ± 897.23	0.345	0.075	0.721
26:0 Lyso PC	1,385.8 ± 653.87	1,102.54 ± 910.66	0.318	903.19 ± 335	947.16 ± 438.03	0.795	0.19	0.721
18:0 Lyso PI	529.75 ± 36.69	515.64 ± 59.52	0.435	492.85 ± 24.4	552.79 ± 213.95	0.391	0.18	0.721
17:1 Lyso PI	1,252.94 ± 178.87	1,089.61 ± 363.22	0.132	984.73 ± 147.55	1,096.62 ± 252.48	0.227	0.014**	0.614
10:0 PE	1,093.63 ± 43.09	1,146.35 ± 264.63	0.482	1,081.86 ± 16.25	1,042.56 ± 167.11	0.415	0.73	0.924
14:0 PE	6,492.53 ± 5,974.79	4,055.62 ± 3,082.77	0.184	4,627.62 ± 3,082.1	4,575.18 ± 2,901.12	0.967	0.79	0.924
15:0 PE	11,785.27 ± 6,093.51	8,547.17 ± 4,909.42	0.072	10,095.38 ± 3,637.19	8,146.27 ± 3,500.89	0.207	0.16	0.721
16:0 PE	988.4 ± 485.91	908.22 ± 683.76	0.705	849.73 ± 427.24	963.64 ± 664.44	0.642	0.81	0.924
18:0 PE	2,034.9 ± 496.09	2,329.83 ± 1,152.16	0.247	2,416.42 ± 667.04	2,722.66 ± 1,096.31	0.446	0.33	0.801
16:0–18:1 PE	1,379.75 ± 789.39	1,576.07 ± 978.96	0.527	1,665.8 ± 591.64	1,486.01 ± 1,083.27	0.642	0.59	0.908
16:0–18:2 PE	5,136.29 ± 2,448.69	5,281.41 ± 2,921.32	0.876	5,561.13 ± 2,147.88	4,789.81 ± 2,123	0.4	0.86	0.924
16:0–20:4 PE	5877.16 ± 3055.62	6060.55 ± 2759	0.847	6847.49 ± 1846.47	5722.44 ± 2030.26	0.185	0.4	0.801
16:0–22:6 PE	14,250.52 ± 9,558.22	11,322.15 ± 5,486.26	0.21	18,820.95 ± 7,779.8	13,656.95 ± 8,840.24	0.159	0.051	0.721
18:0–18:1 PE	161,625.03 ± 79,626.73	168,362.26 ± 97,802.56	0.828	197,295.21 ± 86,481.76	188,582.88 ± 124,196.08	0.852	0.56	0.908
18:0–18:2 PE	69,665.96 ± 34,321.87	75,569.08 ± 45,718.29	0.679	84,184.88 ± 36,961.33	63,384.4 ± 24,117.55	0.118	0.59	0.908
18:0–20:4 PE	155,633.27 ± 116,917.88	164,335.47 ± 88,926.8	0.79	176,382.32 ± 48,553.43	164,588.17 ± 86,657.41	0.704	0.34	0.801
18:0–22:6 PE	7,962.7 ± 4,742.12	6,557.2 ± 3,028.47	0.248	11,067.82 ± 4,138.41	7,685.97 ± 3,895.37	0.058	0.02**	0.614
14:0 Lyso PE	1,915.15 ± 1,566.85	1,209.48 ± 853.36	0.147	1,053.1 ± 288.82	1,183.3 ± 719.29	0.561	0.43	0.801
16:0 Lyso PE	10,796.82 ± 4,459.07	11,985.48 ± 6,465.53	0.551	8,692.55 ± 3,258.92	10,791.91 ± 5,400.7	0.291	0.5	0.891
18:0 Lyso PE	20,833.84 ± 9,790.43	19,921.49 ± 12,047.48	0.811	16,674.49 ± 5,239.81	20,035.85 ± 8,102.26	0.268	0.69	0.924
18:1 Lyso PE	9,765.58 ± 3,171.69	11,603.32 ± 10,078.39	0.526	8,318.33 ± 4,439.21	7,054.24 ± 3,195.95	0.435	0.24	0.721
08:0 PC	2,606.3 ± 755.61	2,729 ± 1,726.91	0.81	2,132.68 ± 683.93	2,157.13 ± 672.23	0.932	0.39	0.801
08:0 PC	2,606.3 ± 755.61	2,729 ± 1,726.91	0.81	2,132.68 ± 683.93	2,157.13 ± 672.23	0.932	0.39	0.801
10:0 PC	1,241.31 ± 223.67	1,254.74 ± 317.56	0.89	1,234.97 ± 131.63	1,110.62 ± 432.8	0.392	0.24	0.721
14:0 PC (DMPC)	12,521.37 ± 4,525.06	12,528.97 ± 5,162.48	1	14,963.49 ± 7,205.62	12,821.7 ± 8,246.37	0.522	0.72	0.924
15:0 PC	10,755.95 ± 3,407.63	10,189.58 ± 4,874.43	0.71	10,506.32 ± 5,695.9	11,063.05 ± 5,199.78	0.809	0.73	0.924
16:0 PC (DPPC)	14,439.61 ± 4,165.07	15,270.96 ± 7,684.05	0.72	15,944.38 ± 3,563.83	17,866.9 ± 7,457.57	0.462	0.4	0.801
17:0 PC	12,377.15 ± 2,150.84	13,175.79 ± 6,545.7	0.553	16,729.1 ± 4,899.13	12,357.96 ± 6,265.94	0.084	0.24	0.721
18:0 PC (DSPC)	4,694.52 ± 1,033.04	5,348.33 ± 2,668.55	0.25	6,553.83 ± 2,302.77	6,303.02 ± 2,581.67	0.811	0.17	0.721
21:0 PC	1,134.68 ± 850.57	727.71 ± 530.73	0.063	664.55 ± 346.71	745.86 ± 804.58	0.769	0.22	0.721
16:1 (Δ9-Cis) PC	12,031.02 ± 6,670.76	10,428.14 ± 5,730.34	0.43	15,984.78 ± 11,296.91	15,337.03 ± 10,538.94	0.889	0.13	0.721
18:1 (Δ6-Cis) PC	468,993.96 ± 75,686.2	432,144.5 ± 205,093.35	0.396	471,269.04 ± 101,844.4	442,254.15 ± 140,306.32	0.588	0.16	0.721
18:2 (Cis) PC (DLPC)	366,805.32 ± 146,307.59	362,169.07 ± 129,003.3	0.92	339,970.68 ± 102,466.54	361,840.58 ± 163,581.43	0.715	0.94	0.957
18:3 (Cis) PC	844.67 ± 513.27	626.57 ± 328.57	0.1	895.7 ± 464.22	1,031.3 ± 431.59	0.478	0.036**	0.721
20:1 (Cis) PC	409.51 ± 162.27	570.04 ± 492.02	0.26	551.04 ± 351.63	609.48 ± 356.27	0.699	0.68	0.924
20:4 (Cis) PC	2,101.76 ± 555.35	2,000.54 ± 737.99	0.66	2,034.62 ± 529.09	2,615.5 ± 1,479.7	0.251	0.35	0.801
16:0–02:0 PC	379.88 ± 50.2	402.8 ± 259.42	0.76	369.67 ± 100.03	314.2 ± 170.89	0.373	0.74	0.924
16:0–18:1 PC	246,113.03 ± 50,809.73	233,797.32 ± 105,608.59	0.69	276,434.05 ± 87,708.37	248,417.23 ± 67,583.13	0.396	0.19	0.721
18:0–22:6 PC	15,807.12 ± 6,498.52	16,339.31 ± 6,210.21	0.8	20,266.62 ± 8,895.09	18,248.53 ± 5,045.87	0.498	0.43	0.801
18:1–18:0 PC	43,882.85 ± 10,977.52	48,632.08 ± 20,248.89	0.33	57,681.16 ± 13,547.17	56,110.69 ± 18,169.87	0.822	0.12	0.721
16:0 SM (d18:1/16:0)	199,072.09 ± 64,400.48	248,256.62 ± 98,543.08	0.107	235,047.52 ± 72,780.35	239,424.32 ± 121,479.08	0.921	0.49	0.883
17:0 SM (d18:1/17:0)	5,003.27 ± 1,830.3	5,074.62 ± 2,525.14	0.927	5,631.89 ± 1,751.27	4,777.45 ± 1,648.23	0.244	0.7	0.924
18:0 SM (d18:1/18:0)	19,254.99 ± 8,146.75	22,804.24 ± 13,582.43	0.388	20,493.61 ± 5,827.37	27,477.25 ± 18,595.39	0.267	0.77	0.924
18:1 SM (d18:1/18:1(9Z))	8,149.24 ± 1,984.27	6,920.97 ± 2,325.88	0.11	7,680.16 ± 1,363.96	8,445.31 ± 2,487.71	0.392	0.091	0.721
24:0 SM	19,358.49 ± 13,424.11	13,592.17 ± 8,958.63	0.11	11,537.64 ± 4,311.3	24,897.51 ± 25,443.34	0.086	0.26	0.744
24:1 SM	79,893.89 ± 29,207.21	84,754.77 ± 37,801.35	0.68	86,917.51 ± 21,746.03	115,850.37 ± 63,863.36	0.148	0.33	0.801
16:0 ceramide	17,444.11 ± 9,068.48	14,405.78 ± 6,811.65	0.23	15,570.62 ± 4,979.85	15,092.58 ± 6,449.51	0.848	0.62	0.914
18:0 ceramide	15,960.54 ± 9,011.1	13,472 ± 5,982.7	0.291	13,506.79 ± 4,699.34	13,268.1 ± 6,014.17	0.919	0.84	0.924
18:1 ceramide	2,258.37 ± 1,006.16	1,822.97 ± 750.54	0.123	1,747 ± 472.81	1,637.6 ± 545.07	0.619	0.29	0.793
20:0 ceramide	1,350.41 ± 422.48	1,275.38 ± 559.87	0.668	1,682.04 ± 800.75	1,507.31 ± 327.27	0.529	0.072	0.721
22:0 ceramide	410.54 ± 141.04	371.57 ± 77.92	0.364	461.45 ± 176.74	445.79 ± 142.5	0.816	0.4	0.801
24:0 ceramide	50,743.61 ± 31,680.82	38,690.65 ± 17,563.71	0.12	49,641.48 ± 28,748.92	40,502.99 ± 14,420.9	0.375	0.55	0.908
24:1 ceramide	7,413.64 ± 2,637.55	7,825.03 ± 3,890.77	0.73	10,396.73 ± 4,494.86	9,168.2 ± 2,595.49	0.454	0.097	0.721

a All values are presented as means ± SD. NN, normal rotator cuff with normal clinical routine serum lipid test results; NA, normal rotator cuff with abnormal clinical routine serum lipid test results; RN, rotator cuff tear with normal routine serum lipid test results; RA, rotator cuff tear with abnormal routine serum lipid test results. Traditional abnormal clinical plasma lipid levels were defined as abnormal levels of at least two of four lipids (TG ≥1.70 mmol/L; TC ≥5.18 mmol/L; L-DLC ≥3.64 mmol/L; HDL-C ≤1.04 mmol/L).

## NN vs. RN, p < 0.05;

** Kruskal-Wallis test based on four groups, p < 0.05. BH-adjusted p-value = Overall p-value adjusted by the Benjamini and Hochberg method.

**Figure 2:**
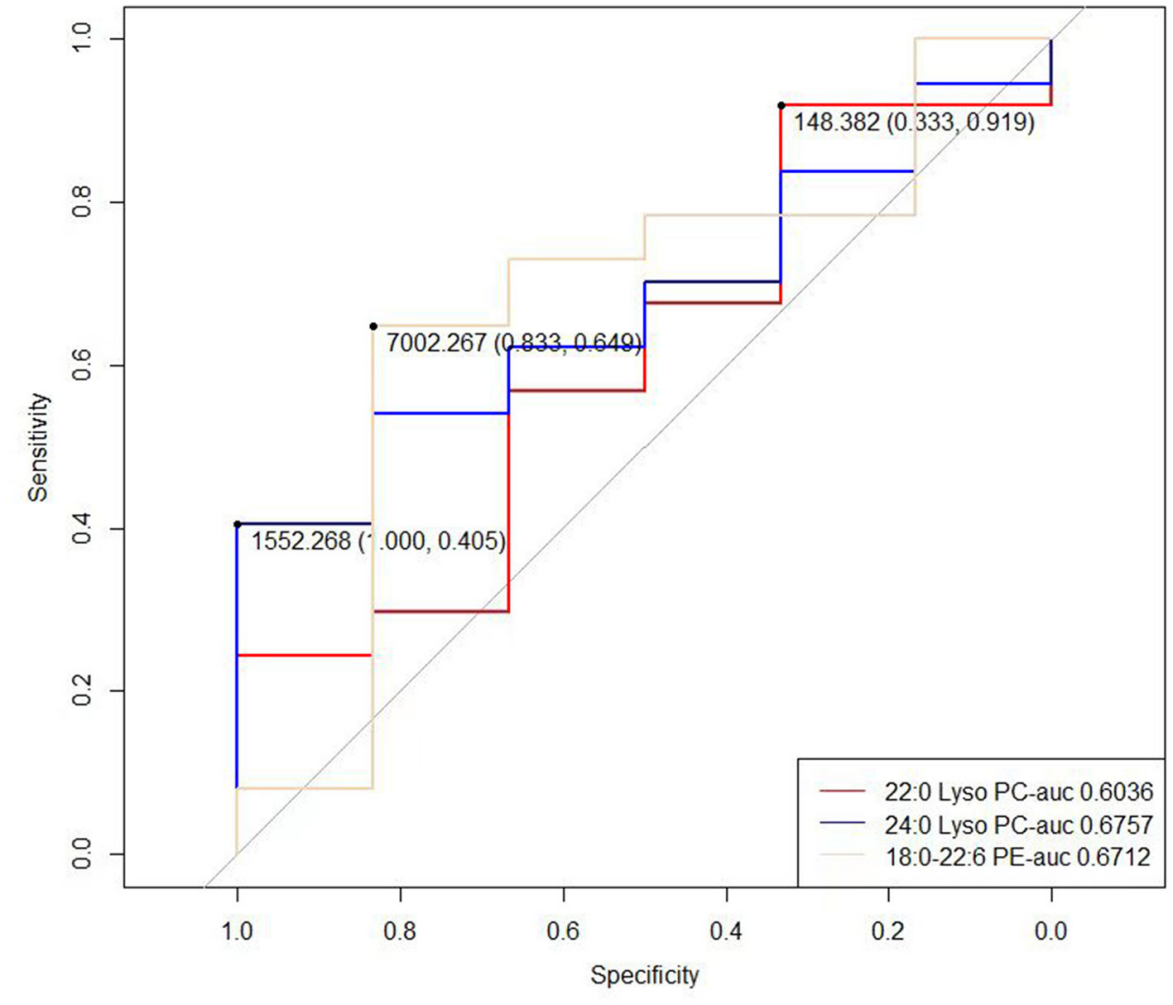
ROC curve of the 22:0 Lyso PC, 24:0 Lyso PC, 17:1 Lyso PI and 18:0–22:6 PE in RN group (rotator cuff tear with normal routine serum lipid test).

In order to determine the possible diagnostic value of our results, we drew ROC curves and calculated AUC values for 22:0 Lyso PC, 24:0 Lyso PC, and 18:0–22:6 PE concentrations in the RN group (rotator cuff tear with normal routine serum lipid test; Figure 2). The AUC values for 22:0 Lyso PC, 24:0 Lyso PC, and 18:0–22:6 PE were 0.6036, 0.6757, and 0.6712, respectively, and the thresholds were 148.382, 1552.268, and 7002.267, respectively. We calculated the sensitivity and specificity of diagnostic methods when using the concentrations of these five metabolites separately (Table 3). According to the results, 22:0 Lyso PC had a highest sensitivity (92%), while 24:0 Lyso PC and 18:0–22:6 PE had the highest specificity (100 and 83%, respectively).

**Table 3:** Sensitivity and specificity of five lipid molecules in the RN group (rotator cuff tear with normal routine serum lipid test).

**Criteria**	**Sensitivity**	**Specificity**	**Accuracy**
22:0 Lyso PC	92%	33%	84%
24:0 Lyso PC	41%	100%	49%
18:0–22:6 PE	65%	83%	67%

### Multivariate statistical analysis results

**Figure 3:**
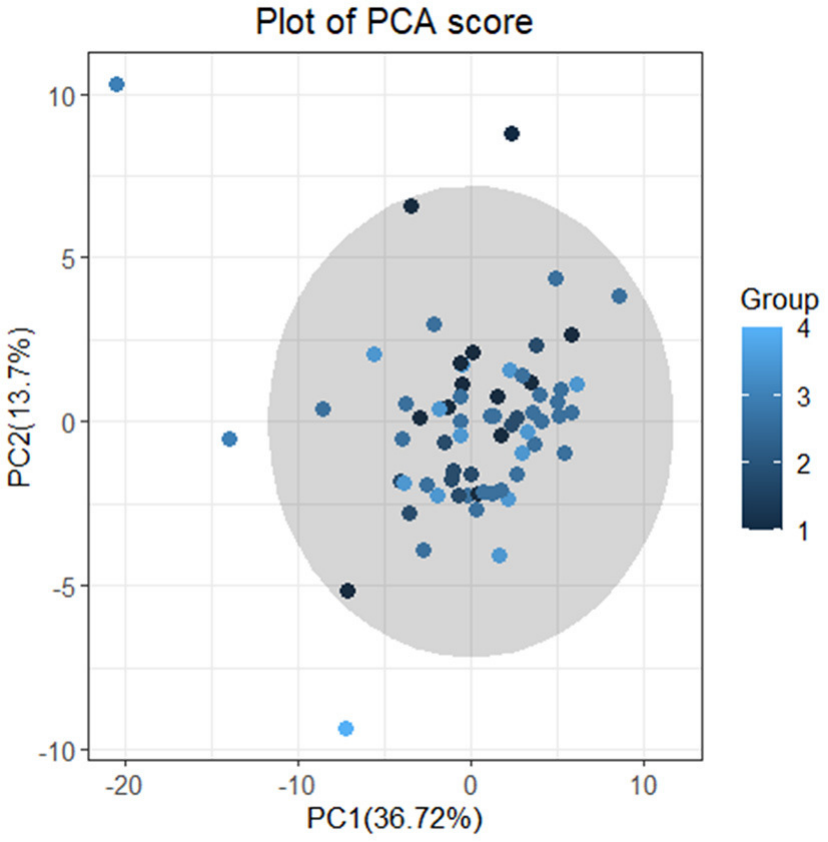
Plot of PCA score. Principal component analysis (PCA). The four groups: group 1 = normal rotator cuff with normal clinical routine serum lipid test results; group 2 = normal rotator cuff with abnormal clinical routine serum lipid test results; group 3 = rotator cuff tear with normal routine serum lipid test results; group 4 = rotator cuff tear with abnormal routine serum lipid test results. Traditional abnormal clinical plasma lipid levels were defined as abnormal levels for at least two of four lipids (TG ≥1.70 mmol/L; TC ≥5.18 mmol/L; L-DLC ≥3.64 mmol/L; HDL-C ≤1.04 mmol/L). The results (PC1 = 36.72% <50%;PC2 = 13.7% <50%) showed that the four groups could not be distinguished according to PCA.

**Figure 4:**
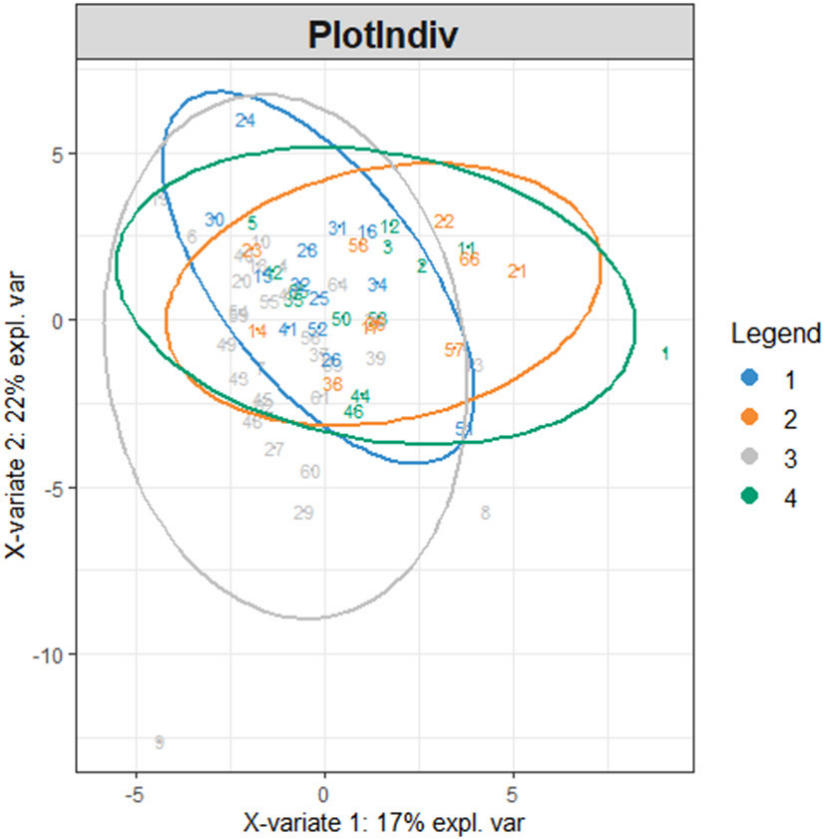
PlotIndiv. The four groups: group 1 = normal rotator cuff with normal clinical routine serum lipid test results; group 2 = normal rotator cuff with abnormal clinical routine serum lipid test results; group 3 = rotator cuff tear with normal routine serum lipid test results; group 4 = rotator cuff tear with abnormal routine serum lipid test results. Traditional abnormal clinical plasma lipid levels were defined as abnormal levels for at least two of four lipids (TG ≥1.70 mmol/L; TC ≥5.18 mmol/L; L-DLC ≥3.64 mmol/L; HDL-C ≤1.04 mmol/L). X-variate 1 and X-variate 2 represent the measured rate of the built model for X and Y matrices, respectively.

Firstly, we conducted principal component analysis (PCA) to observe the overall distribution of each sample and assess the reliability of the whole analysis process. The PCA score plot showed that most of the data for the four groups were included in the 95% confidence interval. However, the results (PC1 = 36.72%, PC2 = 13.7%) showed that the four groups could not be well-distinguished according to PCA (Figure 3). To distinguish the overall differences in lipid metabolism spectrum between groups, a partial least squares-discriminant analysis (PLS-DA) was conducted (Figure 4), and the PlotIndiv results showed that the rotator cuff tear group with normal clinical plasma lipid levels could be distinguished from other groups (X-variate 1 = 17%, X-variate 2 = 22%).

**Figure 5:**
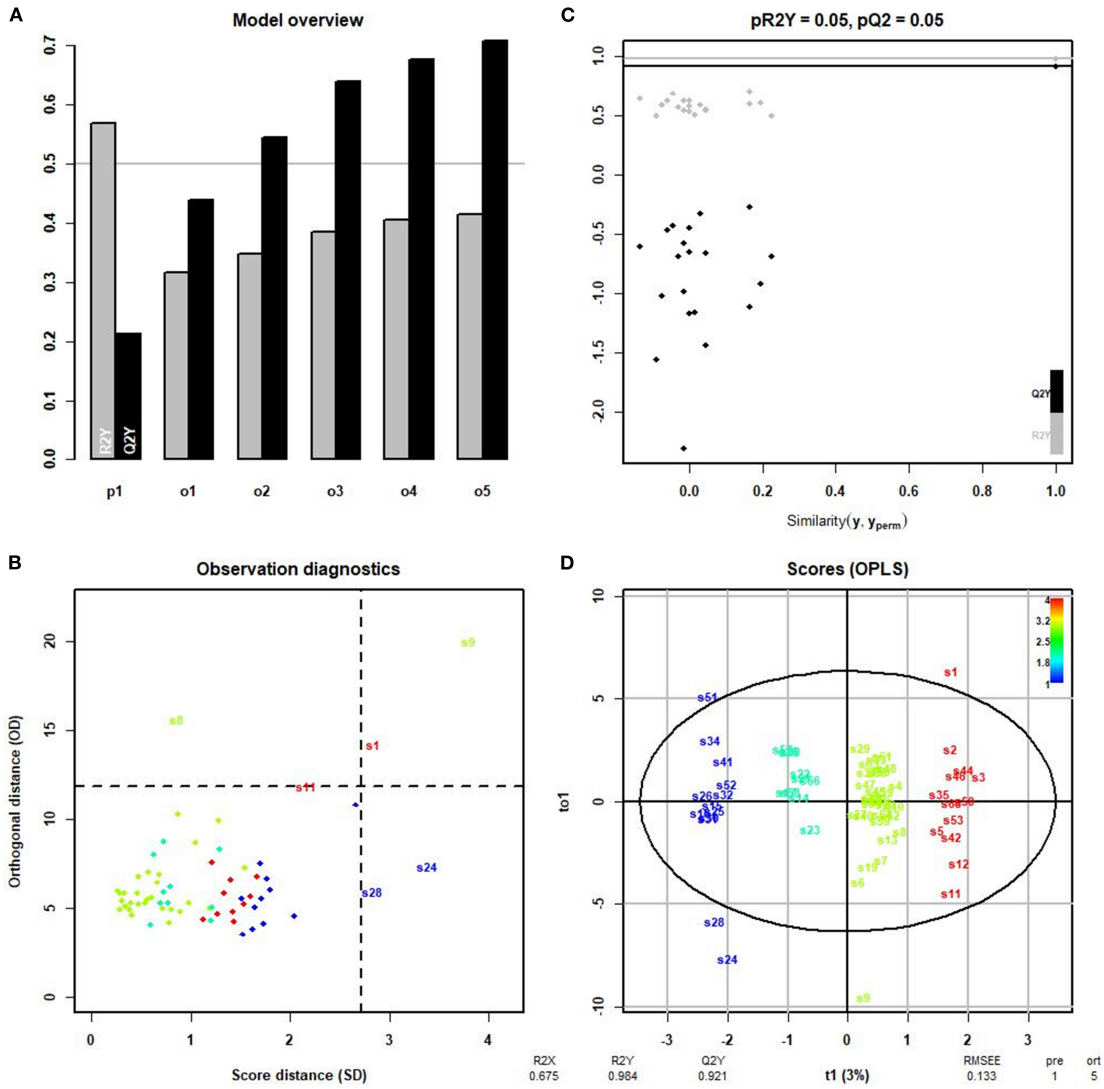
OPLS-DA. **(A)** R2y and Q2y of five orthogonal axes. R2y represents the measured rate of the built model for the Y matrix. Q2y indicates the prediction ability of the model; the closer the value is to 1, the better the fitting degree of the model. **(B)** Samples differing markedly from other samples. S22, 18:0–18:1 PE; S43, 20:4 (Cis) PC; S47, 18:1–18:0 PC. **(C)** Scatter plot showing that the results were stable without overfitting (pR2Y = 0.05, pQ2 = 0.05). **(D)** The four groups: group 1 = normal rotator cuff with normal clinical routine serum lipid test results; group 2 = normal rotator cuff with abnormal clinical routine serum lipid test results; group 3 = rotator cuff tear with normal routine serum lipid test results; group 4 = rotator cuff tear with abnormal routine serum lipid test results. Traditional abnormal clinical plasma lipid levels were defined as abnormal levels for at least two of four lipids (TG ≥1.70 mmol/L; TC ≥5.18 mmol/L; L-DLC ≥3.64 mmol/L; HDL-C ≤1.04 mmol/L). R2x = 0.512; R2y = 0.973; Q2y = 0.879. All values >0.5 indicate that the four groups of samples differed significantly in the OPLS-DA score chart.

We then conducted the orthogonal partial least squares-discriminant analysis (OPLS-DA) to modify the PLS-DA model and maximize the differences between different groups in the model to identify different lipid metabolites in each group. The model overview evaluated the adequacy of orthogonal components based on cumulative interpretation rates, giving R2y and Q2y of five orthogonal axes (Figure 5A). The samples 18:0–18:1 PE, 20:4 (Cis) PC, and 18:1–18:0 PC, which differed greatly from other samples, are shown in Figure 5B. The four groups of samples differed significantly according to the OPLS-DA score chart, and there were no significant differences within each group (Figure 5D). The results showed that R2x, R2y, and Q2y were 0.512, 0.973, and 0.879, respectively. The scatter plot (Figure 5C) for R2y and Q2y values of the original data and simulated models after random arrangement showed that the results were stable without overfitting (pR2Y = 0.05, pQ2 = 0.05).

### Fatty infiltration

Among 43 rotator cuff tear patients, 31 (72.1%) had available MRI layers to assess the fatty infiltration level. Four patients (12.9%) had high fatty infiltration grade and 27 (87.1%) had a low level of fatty infiltration. In patients with a high level of fatty infiltration, abnormal serum lipid levels were observed in two patients (50%), and normal serum lipid levels were observed in 2 patients (50%). For patients with a low level of fatty infiltration, abnormal serum lipid levels were observed in 10 patients (37.0%) and normal serum lipid levels were observed in 17 patients (63.0%). Two lipid metabolites (24:0 SM and 16:0 ceramide) were different in concentration between low- and high-grade fatty infiltration groups (*p* = 0.03634 and 0.017, respectively; Table 4).

**Table 4:** Differences in lipid metabolite concentrations between high-grade and low-grade fatty infiltration groups.

**Lipid metabolite**	***p*-Value**	**Lipid metabolite**	***p*-Value**	**Lipid metabolite**	***p*-Value**
Egg Lyso PC	0.5756	16:0–22:6 PE	0.7458	18:3 (Cis) PC	0.3309
14:0 Lyso PC	0.8366	18:0–18:1 PE	0.7909	20:1 (Cis) PC	0.1849
15:0 Lyso PC	0.7017	18:0–18:2 PE	0.498	20:4 (Cis) PC	0.1329
17:0 Lyso PC	0.8829	18:0–20:4 PE	0.5756	16:0–02:0 PC	0.5756
18:0 Lyso PC	0.8829	18:0–22:6 PE	0.7909	16:0–18:1 PC	0.227
18:1 Lyso PC	0.8366	14:0 Lyso PE	0.3024	18:0–22:6 PC	0.1849
20:0 Lyso PC	0.5756	16:0 Lyso PE	0.3309	18:1–18:0 PC	0.6164
22:0 Lyso PC	0.7458	18:0 Lyso PE	0.7909	16:0 SM (d18:1/16:0)	0.8366
24:0 Lyso PC	0.1329	18:1 Lyso PE	0.7458	17:0 SM (d18:1/17:0)	0.07227
26:0 Lyso PC	0.498	18:0 PC	0.9296	18:0 SM (d18:1/18:0)	0.8366
18:0 Lyso PI	0.6164	10:0 PC	0.5361	18:1 SM (d18:1/18:1(9Z))	0.498
17:1 Lyso PI	0.8829	14:0 PC (DMPC)	0.5756	24:0 SM	0.03643
10:0 PE	0.9765	15:0 PC	0.5361	24:1 SM	0.1488
14:0 PE	0.3024	16:0 PC (DPPC)	0.5536	16:0 ceramide	0.017
15:0 PE	0.1849	17:0 PC	0.1329	18:0 ceramide	0.05546
16:0 PE	0.7909	18:0 PC (DSPC)	0.361	18:1 ceramide	0.1329
18:0 PE	0.2505	21:0 PC	0.3928	20:0 ceramide	0.0634
16:0–18:1 PE	0.498	16:1 (9-Cis) PC	0.227	22:0 ceramide	0.9765
16:0–18:2 PE	0.361	18:1 (6-Cis) PC	0.6585	24:0 ceramide	0.3928
16:0–20:4 PE	0.836	18:2 (Cis) PC (DLPC)	0.7458	24:1 ceramide	0.227

Grading performed according to the degree of fat infiltration following Goutallier's classification (17). An absence of intramuscular fat was classified as stage 0; the presence of some fatty streaks was classified as stage 1; the presence of substantial fat less than the amount of muscle was classified as stage 2; the presence of a quantity of fat roughly equal to the muscle volume was classified as stage 3; the presence of more fat than muscle was classified as stage 4. Stages 0, 1, and 2 were classified as low-grade fatty degeneration, and stages 3 and 4 were categorized as high-grade fatty degeneration.

**Figure 6:**
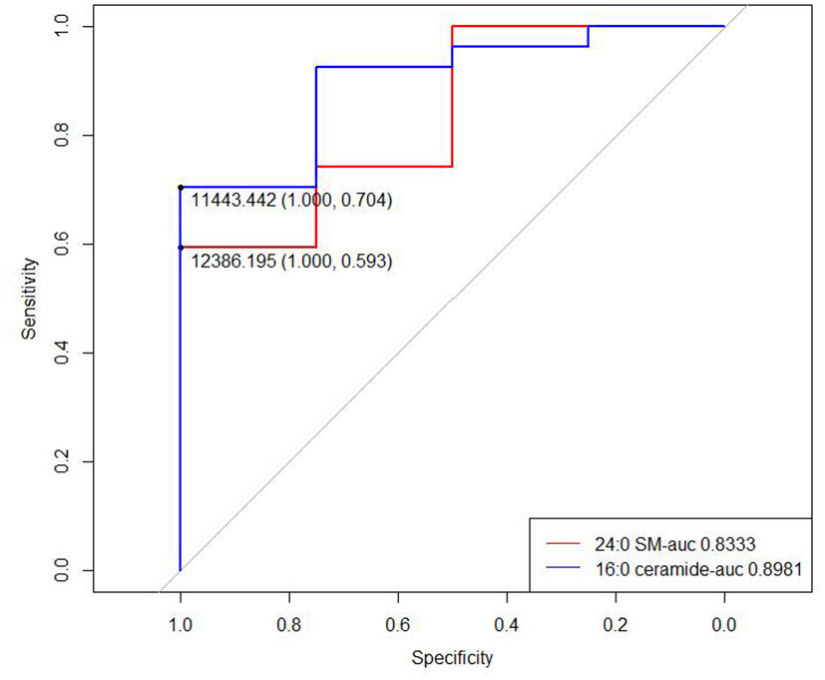
ROC curves of 24:0 SM and 16:0 ceramide for predicting fatty infiltration grade.

We then drew ROC curves and calculated the AUC values of 24:0 SM and 16:0 ceramide concentrations to predict fatty infiltration severity (Figure 6). The AUC for 24:0 SM and 16:0 ceramide was 0.8333 and 0.8981, respectively, and the threshold was 12,386.195 and 11,443.44, respectively. Lower concentrations were observed in high-grade fatty infiltration patients, hence we speculated that these metabolite concentrations can serve as possible diagnostic markers for predicting the severity of fatty infiltration. We calculated the sensitivity and specificity of diagnostic methods when using the concentrations of these two metabolites separately, and also the sensitivity and specificity of the serial tests combing the traditional lipid panel and the two metabolites (Table 5). The sensitivity was 100% for both 24:0 SM and 16:0 ceramide in these populations, higher than for the traditional lipid panel. When using 24:0 SM and 16:0 ceramide as the predicting criteria, specificity was increased compared with using only one of the metabolites. Combining the tradition lipid panel and the metabolites achieved higher accuracy than did other methods.

**Table 5:** Sensitivity and specificity of different criteria for predicting fatty infiltration.

**Criteria**	**Sensitivity**	**Specificity**	**Accuracy**
Traditional lipid panel	50%	63%	61%
24:0 SM	100%	56%	52%
16:0 ceramide	100%	70%	74%
24:0 SM+16:0 ceramide	100%	78%	81%
Traditional lipid panel+24:0 SM	50%	89%	84%
Traditional lipid panel+16:0 ceramide	50%	89%	84%
Traditional lipid panel+24:0 SM+16:0 ceramide	50%	93%	87%

## Discussion

Lipid metabolism disorders play an important role in rotator cuff tear. However, not all patients with rotator cuff tear have traditional abnormal clinical plasma lipid levels. In view of this complicated relationship, it is important to identify potential lipid metabolites that can serve as high-sensitivity markers to facilitate early diagnosis of higher risk or higher severity rotator cuff tear.

Although there were no differences in clinical lipids between rotator cuff tear and normal groups, our results showed significant differences in five lipid molecules, 17:1 Lyso PI, 18:0–22:6 PE, 18:3 (Cis) PC, 22:0 Lyso PC, and 24:0 Lyso PC, between the four groups. Compared with the other three groups, 17:1 Lyso PI and 18:3 (Cis) PC were significantly elevated in the group with normal rotator cuff and normal levels of clinical plasma lipids (NN) and the rotator cuff tear group with abnormal clinical plasma lipid levels (RA). Meanwhile, a significantly lower level of 18:0–22:6 PE was observed in the rotator cuff tear group with normal clinical plasma lipid levels (RN). These five lipid metabolites may contribute to distinguish people with rotator cuff tear from normal people.

Our results may be explained by some articles confirming the effect of lipids on tendons. More than 90% of tendon tissue components are type I collagen, hence the formation of type I collagen is closely related to the structural stability of tendon tissue. Cell density is the critical parameter known to alter both the rates of cell proliferation and the level of cell differentiation, which controls tendon morphogenesis. Tendon morphogenesis is highly dependent on cell density signaling (19). Studies have shown that cell density signal can control the production and accumulation of collagen molecules, and thereby control local collagen deposition. Therefore, cell density signaling plays an important role in tendon injury and repair (20). Petzold and Schwarz (19) proposed the existence of a bone cofactor that regulates the growth of tendon cells through the transmission of cell density signals, and phosphatidylinositol is one of the important components of this cofactor, therefore phosphatidylinositol has an crucial impact on tendon injury and repair (19). Similarly, another study showed that the downregulation of phosphatidylinositol 3-kinase (pi-3k) plays an important role in tendon cell apoptosis, matrix degradation, and inflammation. And phosphatidylinositol is an important component of this heterodimeric lipid kinase (21). These findings are consistent with our results; 17:1 Lyso PI was significantly different between the four groups (*p* < 0.05) based on Kruskal-Wallis tests. This may be one of the reasons for the impaired elasticity and biomechanical stability of tendons in patients with rotator cuff tear.

Our results also showed that in groups with traditional normal clinical plasma lipid levels, 22:0 Lyso PC was elevated and 24:0 Lyso PC was diminished in rotator cuff tear groups compared with normal groups. This result may be attributed to the effects of lysophosphatidylcholine on tendon cells. Previous studies demonstrated that LPC plays an important role in inducing the migration of lymphocytes and macrophages, and increasing the production of pro-inflammatory cytokines, thus inducing oxidative stress, promoting tendon cell apoptosis, and aggravating inflammation, which may promote the occurrence of tendon injury (22). Therefore, regardless of whether clinical blood lipid levels are normal or not, the above-mentioned five lipid molecules may help to screen out people at higher risk of rotator cuff tear, which may contribute to better understanding of risk factors for this ailment, and education to prevent its occurrence.

Although this study did not explore the relationship between clinical blood lipid level and rotator cuff injury, lipidomics, clinical blood lipids, and tendons are closely related, among which hypercholesterolemia is an important risk factor for the occurrence and development of tendon pathology. In recent years, many studies have confirmed the effect of hypercholesterolemia on the musculoskeletal system. In the hypercholesterolemic environment, lipids can accumulate in the extracellular matrix of tendons, resulting in structural, inflammatory, and mechanical changes of tendons, making hypercholesterolemic patients more prone to tendon lesions (23). The results of a prospective study showed that patients with rotator cuff tears were more likely to have hypercholesterolemia (7). Regarding the specific pathological mechanism, some studies have confirmed that hypercholesterolemia is related to the reduction of non-collagen synthesis and the binding of extracellular matrix components, thereby changing the tendon microenvironment (24, 25). In addition, another animal study explored the effect of hypercholesterolemia on the elastic mechanical properties of supraspinatus tendon in mouse, rat, and monkey models. Based on the results, the author believes that in these animals, hypercholesterolemia increases the stiffness and elastic modulus of supraspinatus tendons, resulting in tendon injury (26). According to the review mentioned above, hypercholesterolemia can affect the structure, mechanical properties, and inflammatory changes of tendons, leading to more tendon lesions in patients with hypercholesterolemia.

In addition to the relationship between plasma lipid group and rotator cuff injury, the relationship between lipid group and continuous fat infiltration pathology is also very important. We believe that some lipid molecules may affect the severity of rotator cuff injury by causing metabolic disorders and dyslipidemia. A study conducted by Bhattacharyya et al. (27) showed that lipids found within xanthomas are derived from circulating plasma rather than synthesized, revealing a close connection between serum lipids and local fatty infiltration (27). Thus, we further focused on the relationship between fatty infiltration levels and lipid metabolism. According to a previous study on a rabbit model, hypercholesterolemia was shown to correlate with a deleterious effect on fatty infiltration (15). However, the exact influence of serum lipid level on rotator cuff fatty infiltration in patients is still unclear. Based on existing data sources, we further investigated the relationship between lipid metabolite concentrations and rotator cuff fatty infiltration severity. In our study, two lipid metabolites (24:0 SM and 16:0 ceramide) showed insignificant but measureable differences in concentration, with a *p* > 0.05 but <0.1. Besides these two molecules, concentrations of another three lipid metabolites, namely 17:0 SM (d18:1/17:0), 18:0 ceramide, and 20:0 ceramide, showed no statistically significant differences at *p* < 0.1. Although no exact relationship was found, further research with a larger sample size is needed to explore the possible connection between lipid metabolite concentrations and fatty infiltration. The mechanism of fatty accumulation at the injury site may be further explained by exploring the physiological functions of these lipid molecules.

Studies have also shown that hyperlipidemia is a common risk factor for cardiovascular and tendon diseases (28). Identifying predictive factors of hyperlipidemia may not only provide hints for tendon diseases, but also indicate the serious consequences caused by cardiovascular diseases. In the context of tendon injury, active intervention in hyperlipidemia can indirectly reduce the risk of cardiovascular disease and other serious diseases at the same time.

The present study has some limitations. First, due to the small sample size, it is difficult to identify weak correlations. Therefore, future studies using a larger dataset should be conducted. Second, the results should be taken with caution, since only participants from China were included, and extrapolation to the other populations might not be directly applicable. Third, the best MRI layer (the standard Y-view) to assess fat infiltration using Goutallier's classification is the outermost level, where the scapula joins the scapula body. However, this layer is much more buried than the rotator cuff insertion layer, and this layer is typically missed when collecting images. The adjacent areas of the standard layer can partially suffice for assessing fatty degeneration level, hence we included the contiguous two layers to amplify the sample volume. However, despite this approach, only 72.1% of patients had available MRI layers to assess, resulting in an obvious risk of bias. The number of patients with high-grade fatty infiltration rotator cuff was also relatively small, which means comparison between high- and low-grade fatty infiltration groups may be statistically unreliable. Further research with a larger sample volume is clearly needed.

## Conclusion

In summary, we discovered that five lipid molecules, 17:1 Lyso PI, 18:0–22:6 PE, 18:3 (Cis) PC, 22:0 Lyso PC, and 24:0 Lyso PC, are closely related to rotator cuff tear based on two statistical analysis methods, independent of clinical routine serum lipid test results. This indicates that lipidomics assays are more sensitive than conventional lipid tests, and more suitable for studying rotator cuff lipid metabolism. In addition, two lipid metabolites, 24:0 SM, and 16:0 ceramide, were found to be potential markers of severe fat infiltration. These findings provide insight into how metabolic mechanisms associated with dyslipidemia affect rotator cuff disease.

## Data availability statement

The raw data supporting the conclusions of this article will be made available by the authors, without undue reservation.

## Ethics statement

The studies involving human participants were reviewed and approved by the Ethics Committee of Peking University Third Hospital. The patients/participants provided their written informed consent to participate in this study. Written informed consent was obtained from the individual(s) for the publication of any potentially identifiable images or data included in this article.

## Author contributions

All authors listed have made a substantial, direct, and intellectual contribution to the work and approved it for publication.

## Funding

This work was supported by grants from the National Natural Science Foundation of China (Grant Nos. 82172447 and 81871761) and the Beijing Natural Science Foundation (Grant No. 7192221).

## Conflict of interest

The authors declare that the research was conducted in the absence of any commercial or financial relationships that could be construed as a potential conflict of interest.

## Publisher's note

All claims expressed in this article are solely those of the authors and do not necessarily represent those of their affiliated organizations, or those of the publisher, the editors and the reviewers. Any product that may be evaluated in this article, or claim that may be made by its manufacturer, is not guaranteed or endorsed by the publisher.
